# The Fast and One-Step Growth of ZnO Nanorods on Cellulose Nanofibers for Highly Sensitive Photosensors

**DOI:** 10.3390/nano13182611

**Published:** 2023-09-21

**Authors:** Naveed ul Hassan Alvi, Mohammad Yusuf Mulla, Tiffany Abitbol, Andreas Fall, Valerio Beni

**Affiliations:** 1 Smart Hardware, RISE Research Institutes of Sweden, Bio- and Organic Electronics, Södra Grytsgatan 4, Plan2, 602-33 Norrköping, Sweden; 2 Digital Cellulose Center, 602-33 Norrköping, Sweden; 3 Smart Materials, RISE Research Institutes of Sweden, Bioeconomy & Health, Drottning Kristinas Väg 61B, 114-28 Stockholm, Sweden

**Keywords:** zinc oxide, cellulose nanofibers, photosensors, hydrothermal growth, highly photosensitive

## Abstract

Cellulose is the most abundant organic material on our planet which has a key role in our daily life (e.g., paper, packaging). In recent years, the need for replacing fossil-based materials has expanded the application of cellulose and cellulose derivatives including into electronics and sensing. The combination of nanostructures with cellulose nanofibers (CNFs) is expected to create new opportunities for the development of innovative electronic devices. In this paper, we report on a single-step process for the low temperature (<100 °C), environmentally friendly, and fully scalable CNF-templated highly dense growth of zinc oxide (ZnO) nanorods (NRs). More specifically, the effect of the degree of substitution of the CNF (enzymatic CNFs and carboxymethylated CNFs with two different substitution levels) on the ZnO growth and the application of the developed ZnO NRs/CNF nanocomposites in the development of UV sensors is reported herein. The results of this investigation show that the growth and nature of ZnO NRs are strongly dependent on the charge of the CNFs; high charge promotes nanorod growth whereas with low charge, ZnO isotropic microstructures are created that are not attached to the CNFs. Devices manufactured via screen printing/drop-casting of the ZnO NRs/CNF nanocomposites demonstrate a good photo-sensing response with a very stable UV-induced photocurrent of 25.84 µA. This also exhibits excellent long-term stability with fast ON/OFF switching performance under the irradiance of a UV lamp (15 W).

## 1. Introduction

Cellulose is the essential component of plant fibers (60–70% of their solid content). It is an abundant, sustainable, and renewable biopolymer with exceptional mechanical and chemical stability under normal atmospheric conditions. These properties have made cellulose a key material in our daily life (e.g., in paper and packaging). Apart from the traditional paper and packaging industries, recently cellulose and its nano-derivatives have gained a lot of interest in areas such as the food industry and in biomedical applications [1,2,3]. A significant trend in the last few years is towards exploring cellulose and its derivatives for electronic applications [4,5] including the development and exploitation, in device manufacturing, of nanocellulose and inorganic/organic nanostructured materials composites [6].

Cellulose nanofibers are isolated from native cellulose sources via a mechanical treatment that is usually preceded by a chemical or enzymatic pretreatment that has the practical effect of lowering the energetic input required to delaminate the original cellulose fibers [7]. Generally, CNFs are polydisperse, with their dimensions depending on the source material from which they are derived, the type of pretreatment applied, and the extent of the mechanical delamination. Without size fractionation, a single CNF preparation contains small fibrils with widths of a few nanometers and lengths in the hundred-nanometer scale, large fibrils with micrometer scale dimensions, and all sizes in between. Recently, CNFs have attracted considerable interest and have motivated researchers and scientists to develop environmentally friendly cellulose-fiber-based functional materials to develop advanced electronic devices. CNFs can be utilized as an independent functional material or as a supporting component to fabricate nanocomposite materials [8]. Furthermore, CNFs have unique morphology and excellent structural properties, including a large number of surface-reactive groups, which enable modification through covalent bonding, physical adsorption, or surface graft polymerization to further enhance performance [1,9,10,11]. Recently, many articles have been published on the production, modification, and application of CNFs ([8] and references therein). Different methods were adopted for the fabrication of CNF/CNC composites with inorganic nanomaterials, i.e., sacrificial templating, precursor, electrospinning, layer-by-layer deposition, liquid-crystal templates, co-templating, sol-gel, and foaming techniques [12]. Functionalized CNFs have the potential for practical application in the fabrication and development of a large variety of electronic devices such as chemical sensors, biosensors, UV sensors, strain sensors, actuators, transistors, light-emitting diodes, photoelectrodes, solar cells, charge-storing batteries, and piezoelectric energy generators [13,14,15,16].

ZnO is an exceptional semiconductor material with a direct wide bandgap of (3.4 eV). It has outstanding semiconducting properties with large electron–hole binding energy (60 meV) and excellent electron mobility (1000 cm^2^ V^−1^·s^−1^ for a single nanowire). It is highly photosensitive and has the best conductivity for both electrons and holes. ZnO nanostructures have high physical and chemical stability under rough conditions. It is an inexpensive, abundant, and non-toxic green material [17,18,19,20,21].

ZnO nanostructures can be fabricated through a simple, low-temperature (<100 °C) and cost-effective scalable fabrication methods with a large variety of sizes and shapes (nanocombs, nanowires, nanorings, nanohelixes, nanotubes, nanowalls, nanobows, tetrapods, nanoflowers, nanocages, nanobelts, etc.) [22,23], which suggest that this is a promising material which has the full capability to be utilized in the fabrication of different opto-electronics devices, like ultraviolet photodetectors and photocatalytic/photovoltaic devices [24,25,26], piezoelectric nanogenerators [27,28], sun-blocking screens, miniaturized lasers, sensors, transparent electronics/electrodes [29], and light-emitting diodes [30].

The direct growth of zinc oxide NRs on the surface of CNFs (utilizing native negative charges on the surface of CNFs through electrostatic bonding) is an appealing approach [31] and will open up many new opportunities to utilize this nanocomposite material for application in electronic devices. In this work, we have grown highly dense ZnO NRs on CNFs through a simple one-step aqueous chemical approach. We have investigated the possibility of utilizing these functionalized CNFs to develop a large variety of printable and adaptable electronic devices.

## 2. Experimental Section

### 2.1. Synthesis of Cellulose Nanofibers with Different Degrees of Substitution

All CNFs were prepared from a dissolving grade pulp (Domsjö-MORE). Enzymatic grade CNFs were produced as described earlier by Henriksson et al. [32]. The microfluidization to liberate the CNFs from the enzymatically pretreated pulp was conducted through 3 passes at 400 bar, followed by 5 passes at 1700 bar, using a M110-EH microfluidizer (Microfluidics). Enzymatic CNFs have a small negative charge of ~30 µeq/g. Carboxymethylated CNFs were produced via microfluidization (1 pass, 1700 bar) of a carboxymethylated pulp, prepared according to a general protocol described by Wågberg et al. [33] and following [34] to obtain a higher degree of substitution (DS). Two different degrees of substitution (DS) were prepared, DS 0.1 and DS 0.3, corresponding to 600 and 1600 µeq/g, respectively, of charge density.

### 2.2. Synthesis of Zinc Oxide Nanorods on Cellulose Pulp Fibers

The ZnO NRs were fabricated on the surface of the cellulose nanofibers, following a hydrothermal process previously described by Vayssieres et al. [35]. The synthesis solution was prepared by mixing an equimolar concentration (0.1–0.75 M) of zinc nitrate hexahydrate (Zn(NO_3_)_2_ 6H_2_O) and hexamethylene-tetramine (HMTA, C_6_H_12_N_4_). Finally, the CNFs (1% in water) were mixed in the growth solution and were stirred well using magnetic stirring. Finally, the container was left in a microwave oven at 700 W for 90 s. The growth of ZnO NRs on CNFs follows the reactions in [36].

Initially, the reaction between HMT (C_6_H_12_N_4_) and water generates ammonia,

(CH_2_)_6_ N_4 (s)_ + 6H_2_O _(l)_ → 6HCHO _(g)_ + 4NH_3 (g)_ (1)

The ammonia produced in the above step reacts with water and produces ammonium and hydroxide ions,

NH_3 (g)_ + H_2_O _(l)_ → NH^4+^ + OH^−^ (2)

Finally, hydroxide ions react with zinc ions to produce zinc hydroxide and then the decomposition of zinc hydroxide starts the synthesis of solid ZnO NRs on the surface of cellulose nanofibers, under heating.

2OH^−^ + Zn^2+^ _(S)_ → Zn(OH)_2 (S)_ → ZnO_(s)_ + H_2_O _(l)_ (3)

Following the growth step, the produced ZnO NRs/CNF nanocomposites were removed from the growth solution and kept in fresh DI water and the remaining solution was removed using a simple filtration process.

### 2.3. Electrode Printing

The interdigitated carbon electrodes (IDE) (with an area of 0.5 cm^2^) were screen-printed using a screen printer (DEK Horizon 03iX, Munich, Germany) on the paper substrate using a commercially available carbon ink (7102 from DuPont, Wilmington, DE, USA) and a specially designed screen-printing mask. The electrodes were dried in a conventional oven at 120 °C for 5 min. The printed electrodes were further employed for sensor fabrication.

### 2.4. Drop Casting of Zinc Oxide/Cellulose Nanofibers on Printed Carbon Electrodes

The mixture of water and ZnO NRs nanofibers was drop-cast on printed carbon electrodes. The sensor was dried at 100 °C for 10 min in the oven. The printed electrodes were used to measure the photosensitivity and humidity sensitivity of the ZnO NRs/CNF.

### 2.5. Measurements

The morphological, structural, and elemental characterizations of the ZnO NRs/CNF nanocomposites were achieved using a scanning electron microscope (SEM, Sigma 500 Gemini equipped with EDS). The SEM images were captured using the in-lens detector that typically collects secondary electrons and provides topographical information about the sample at magnifications of 250× to 1,000,000. The working distance was 5.0 mm with a voltage (ETH) of 1.0 kV. Topographical imaging of CNF DS0.1 and CNF DS0.3 were performed using a MultiMode 8 atomic force microscope (Bruker, Santa Barbara, CA, USA) in PeakForce tapping mode. A cantilever from Bruker with a nominal spring constant of 0.4 N/m and a nominal tip radius of 2 nm was used. The samples were prepared by spin-coating dilute suspensions on freshly cleaved and PAH-coated mica. The Fourier-transform infrared spectroscopy (FTIR) investigations were performed using an FTIR spectrometer (Bruker Equinox 55, Billerica, MA, USA). The measurements were performed in the transmission mode using a DTGS detector at room temp. in the wavenumber range of 370–4000 cm^−1^. The samples were deposited on transparent potassium bromide (KBr) substrates.

The photoconductive and ON/OFF switching measurements of the fabricated devices were carried out at the photonics lab at ITN, Linköping University, Sweden. The fabricated devices were optically excited using a 15 W UV lamp (ʎ = 365 nm Dutscher, Model E2113, Bernolsheim, France) while the photocurrent was measured using a Keithley 2400 source meter. The experimental data were collected and analyzed using OriginLab software (version 2020 64-bit). For the demonstration of the device as a UV sensor, a 10 W UV lamp (ʎ = 380, Phoseon Technology, Fire Edge FE410, Hillsboro, OR, USA) was used as a light source.

## 3. Results and Discussions

**Figure 1:**
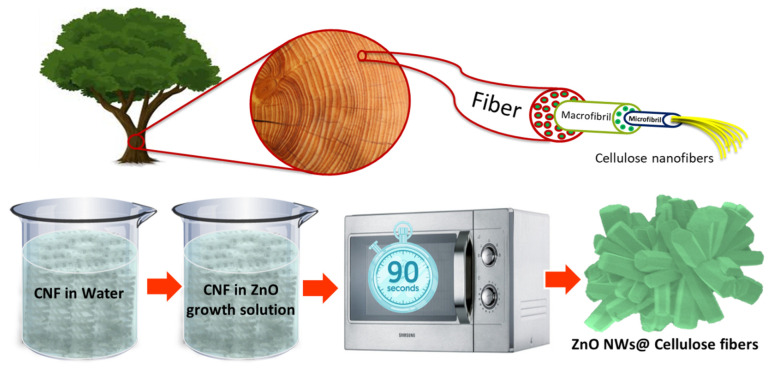
Schematic illustration of extraction of CNFs from tree macrofibril to microfibril and nanofiber levels. Schematic illustration the single-step growth of ZnO NRs on cellulose nanofibers. SEM picture of a single cellulose nanofiber decorated with ZnO NRs is also shown.

Figure 1 presents a schematic illustration of the extraction process of CNFs from tree macrofibril to microfibril and nanofibril levels. The journey of the device from tree to wood tissues to macrofibril and nanofibers is normally completed through several mechanical and chemical procedures. Specifically, wood chips are thermochemically treated via a well-known bleaching chemistry to remove lignin and enrich the cellulose content. To fibrillate cellulose fibers and yield nanofibers, the resulting wood pulp is usually treated enzymatically to loosen the fiber structure or chemically to graft charge groups onto the fibers that lead to repulsive interactions, both tactics that encourage efficient fibrillation via mechanical means. In the current work, the pulp was carboxymethylated to different extents (resulting in the different degrees of substitution/DS) followed by a mechanical treatment consisting of high-pressure microfluidization to yield the cellulose nanofibers of different DS [37]. This work also illustrates the facile and single-step growth procedure of ZnO NRs on CNFs using a domestic microwave oven. The SEM image of a single cellulose nanofiber decorated with ZnO NRs is shown, where nanocellulose fiber is fully covered with ZnO NRs. The CNFs were decorated with ZnO NRs through a fast (90 s), low-temperature (~90 °C), and simple (one-step) aqueous chemical growth method.

**Figure 2:**
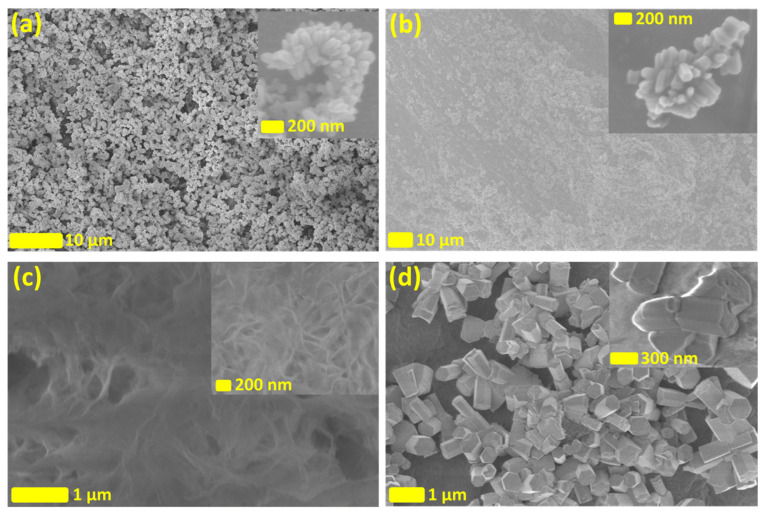
The SEM images of the ZnO NRs grown on the CNFs with different surface charge densities (**a**) CNFDS0.3, (**b**) CNFDS0.1, and (**c**) CNF Enzymatic, and (**d**) shows the freely grown ZnO microstructures.

In Figure 2, the scanning electron microscopic (SEM) morphological and structural investigation of the ZnO NRs grown on the surface of the CNFs is shown. CNFs with different surface charge densities (a) CNFDS0.3 (high), (b) CNFDS0.1 (medium), and (c) CNF Enzymatic (low), affect the surface coverage of ZnO NR growth. The obtained structures have hexagonal wurtzite structures confirming typical ZnO NR formation. The CNFDS0.3 nanofibers are fully covered with ZnO NRs while CNFDS0.1 nanofibers are partially covered and there is almost no growth of ZnO NRs on the enzymatic nanofibers. It is evident that the negative charges introduced on the surface of CNF via the carboxymethylation process are key for the growth of ZnO NRs on the surface of cellulose nanofibers. The negative surface charge provides nucleation for the growth and integration of ZnO NRs. The average estimated diameter of the grown ZnO NRs is about 50–80 nm and the average length is about 200–300 nm. The clusters of the ZnO NRs on individual fibers of CNF are clearly visible, while the ZnO NRs show no successive branching, confirming the high crystallinity and uniform growth. The high aspect ratio growth of ZnO NR with a smooth and uniform surface having fixed diameter is observed. This confirms the faster growth of NRs throughout the c-axis with high anisotropy. Figure 2d displays the SEM images of the free-grown ZnO microstructures; these structures grow as a byproduct in the growth solution when enzymatic nanofibers are used. In the aqueous chemical growth method when no growth sites/templates are available it constructs free microstructures in the solution; examples of these structures are shown in Figure 2d.

**Figure 3:**
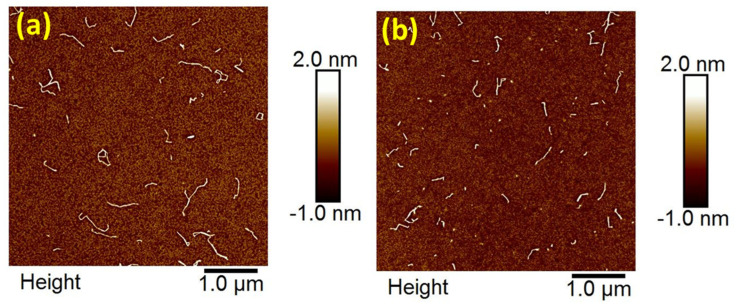
The AFM images displaying CNF fibers on polyallylamine hydrochloride (PAH)-coated mica. In (**a**) CNFDS0.1 (medium-charge-density) and in (**b**) CNFDS0.3 (high-charge density).

Figure 3a,b displays the atomic force microscopic (AFM) images of two different degrees of substitution (DS) of carboxymethylated CNFs CNFDS0.1 (medium charge density) and CNFDS03 (high charge density), respectively. It indicates the diameter of nanofibers is reduced to the nanoscale ~2 nm but the length is in the range of ~100 nanometers to 1 micron.

**Figure 4:**
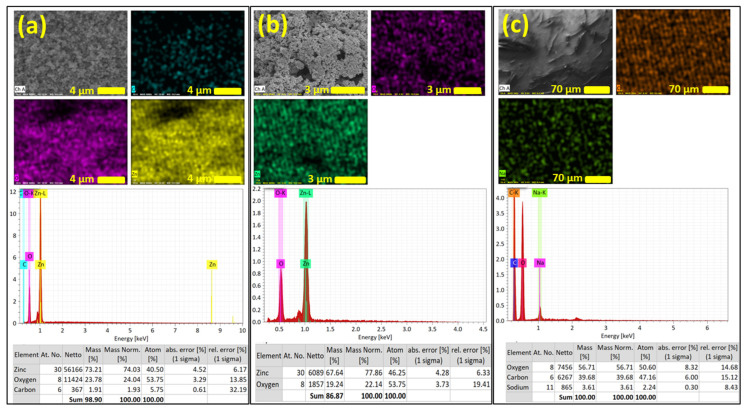
SEM images and the corresponding elemental distribution map obtained with EDS (**a**) ZnO NRs on CNFs, (**b**) free-grown ZnO microstructures, and (**c**) CNF. It also shows the EDS spectrum and a table with elements, mass and atom percentage for all samples.

Elemental information about the ZnO NRs on CNF is obtained using scanning electron microscopy-energy dispersive X-ray spectroscopy (SEM-EDS). Figure 4a shows the SEM image of ZnO NRs grown on CNFs where the corresponding relative distribution mapping of carbon, oxygen, and zinc species over the surface of the coated substrate is obtained with EDS. The uniform distribution of zinc over the sample is observed, confirming the uniform coverage of ZnO NRs. The EDS spectrum of ZnO NRs on CNFs and the table shows the relative elemental mass percentage of zinc as 73.2%, oxygen 23.7%, and carbon 1.9%. This confirms the presence of carbon, oxygen, and zinc. The EDS analysis reveals that the fabricated nanowires are composed of zinc and oxygen and the carbon belongs to the CNF fibers. Figure 4b shows the SEM images of the free-grown ZnO microstructures where the corresponding relative distribution mapping of zinc and oxygen species is displayed. The EDS spectrum of ZnO microstructures and the table show the relative elemental mass percentage of zinc as 77.86% and oxygen 22.14%. This confirms the presence of zinc and oxygen in the sample. It reveals that the fabricated microstructures are composed of zinc and oxygen only. Similarly, Figure 4c displays the SEM images of CNF where the corresponding relative distribution mapping of oxygen, carbon, and sodium species is displayed. The EDS spectrum and the table show the relative elemental mass percentage of oxygen as 56.71%, carbon 39.68%, and sodium 3.61%. This confirms the presence of oxygen, carbon, and sodium in the sample. It reveals that a small amount of sodium is present in CNF.

**Figure 5:**
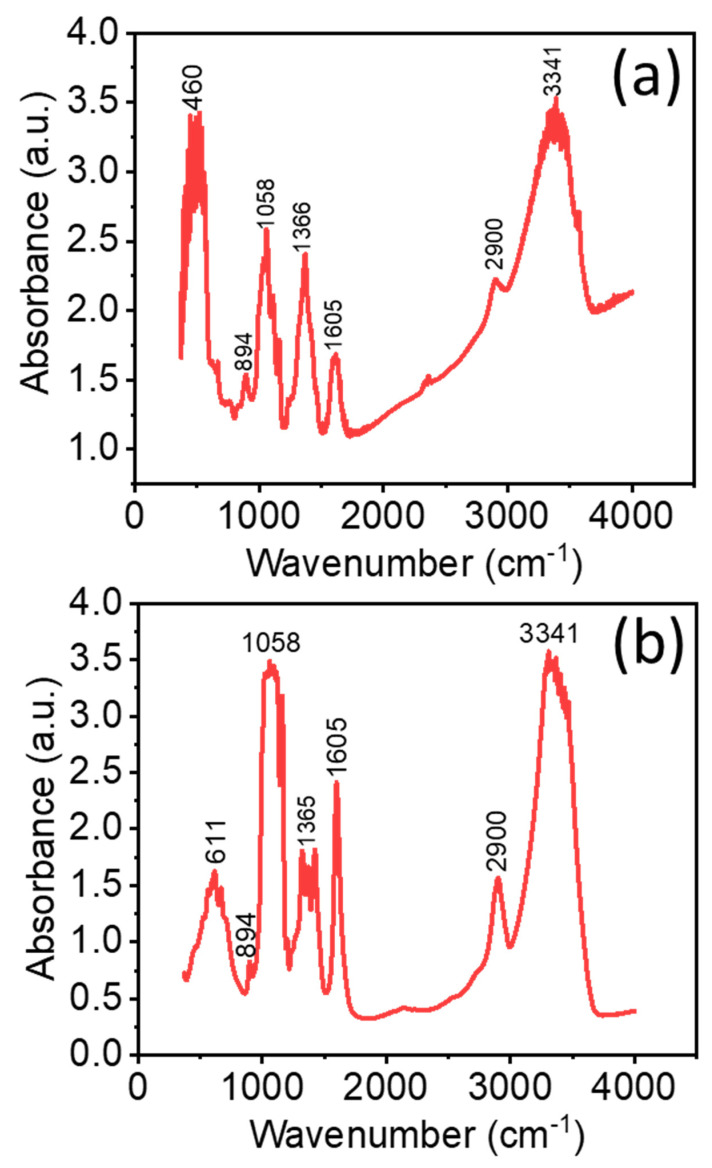
FTIR spectra of (**a**) ZnO NRs/CNF and (**b**) CNF film in the transmission mode.

Figure 5 shows FTIR spectra of (a) ZnO NRs/CNF and (b) CNF film in the transmission mode. The peaks at 3341 cm^−1^ and 1365 cm^−1^ are related to the bending and/or stretching vibrations of (OH^-^) hydroxyl groups [38]. The peak observed at 1058 cm^−1^ is related to the stretching of C-OH [39]. A broad peak centered at 460 cm^−1^ with shoulders from 373 to 577 cm^−1^ is in good agreement with the reported region of metal oxides. It is assigned to the Zn-O stretching vibration which confirms the nanocrystalline structure of the grown ZnO NRs [31,40,41]. The peak observed at around 1605 cm^−1^ relates to the stretching mode of C-O and C=O [42]. The peak observed at 1366 cm^−1^ corresponds to the CH bending vibration in cellulose [43]. It is observed that the peaks centered at 460 cm^−1^ and 3341 cm^−1^ are saturated at the detection limit of the detector, however, it will not change our conclusions since all peaks in the region of 350–600 cm^−1^ correspond to the metal oxides (ZnO) metal-oxygen (M-O) band while those in the region around 3341 cm^−1^ are related to the bending and/or stretching vibrations of (OH^−^) hydroxyl groups (in this case the CNF). Furthermore, the use of less material to avoid saturation at the limit of the detector risks losing small peaks in the spectrum.

**Figure 6:**
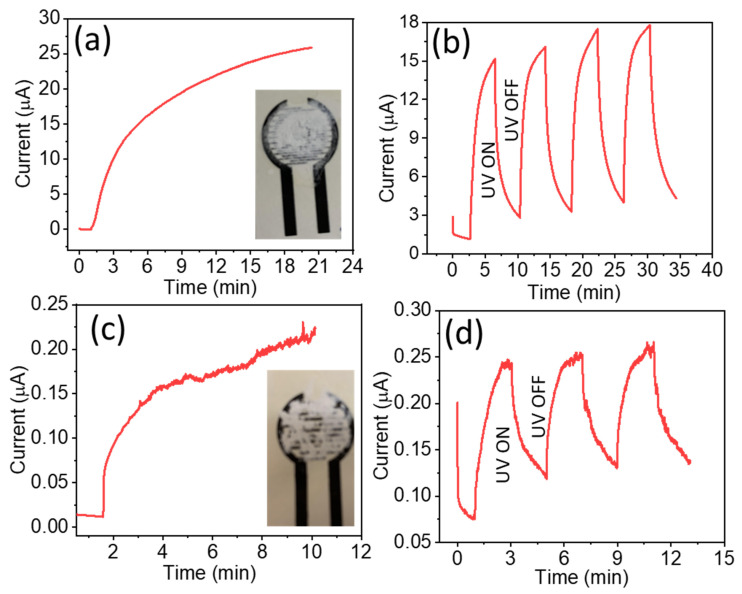
Photo-response and ON/OFF switching performance of the ZnO-NRs@CNF devices under an irradiance of UV light, (**a**,**b**) shows the devices with CNFDS0.3 (with high charge density on the surface) device and (**c**,**d**) with CNFDS0.1 (medium charge density), respectively. Pictures of the fabricated devices are displayed in the inset, where ZnO NRs/CNF was deposited on the printed carbon electrodes on a paper substrate through a simple drop-casting method.

The UV photo-response and ON/OFF switching performance of the ZnO NRs/CNF devices under an irradiance of UV light is shown in Figure 6. The difference in the UV response of the device manufactured with nanocomposites from the growth reaction containing CNFDS0.3 is shown in Figure 6a,b and Figure 6c,d for CNFDS0.1, respectively. All experiments were performed under the irradiance of a UV lamp (15 W). The photo response for ZnO NRs/CNF (CNFDS0.3) was measured for a long period of time (20 min) at a constant biasing of 1V as shown in Figure 6a. It displayed a relatively stable photocurrent of 25.84 µA while ZnO NRs/CNF (CNFDS0.1) displayed low photocurrents of 0.22 µA with poor stability for a long time as shown in Figure 6c, respectively. It shows that ZnO NRs/CNF (CNFDS0.3) devices have strong and steady interconnectivity to display a relatively stable photocurrent for a long time while ZnO NRs/CNF (CNFDS0.1) devices have poor and shaky interconnectivity and display a very low (~100 times less) and unstable photocurrent for a long time.

The dynamics of the ON/OFF switching performances of the ZnO NRs/CNF (CNFDS0.3) device is shown in Figure 6b. It was measured under the irradiance of a 15 W UV lamp by applying a UV light pulse (ON/OFF) for 3 min. The photocurrent pulse shows very good stability and reversibility with a fast rise/fall under a continuous ON/OFF switching of UV light. The photoconductive sensitivity of the device can be determined using the following relation,

$$\mathrm{Photoconductive}\mathrm{sensitivity}=\frac{I_{\max}-I_{\min}}{I_{min}}$$

The device has a sensitivity of 4.37, where *I_max_* = 15.16 µA and *I_min_* = 2.82 µA.

The fabricated ZnO NRs/CNF devices exhibit a small dark current which relates to the development of an electron depletion region near the surface of the ZnO NRs. In the dark, the oxygen molecules in the atmosphere adsorb on the surface and attract free electrons from ZnO NRs to generate oxygen ions (O_2_^−^) and these oxygen ions are in fact responsible for the dark current in the fabricated devices, as described below,

O_2_ (g) + e^−^ → O_2_^−^ (ad)

When the fabricated devices were illuminated with UV light, the photons are captured in ZnO NRs and create electron–hole pairs. The photogenerated holes were easily captured by negative oxygen ions on the surface,

h^+^ + O_2_^−^ (ad) → O_2_ (g)

and photogenerated unpaired electrons are freely available to generate the photocurrent [24,26]. The surface area is a key parameter for generating the photocurrent. The NRs’ structures with several orders of magnitude more surface area (compared to thin film) are the highly preferred structures to efficiently harvest the photons, allowing for the development of highly efficient and cost-effective photodetectors. The ON/OFF switching performances of the ZnO NRs/CNF (CNFDS0.1) device is shown in Figure 6d. Photocurrent pulse shows comparatively less stability and reversibility with slow rise/fall under a continuous ON/OFF switching of UV light. The slow transition time in ZnO nanostructures can be attributed to the surface defects related to electron-trapping regions. It is also reported that O_2_ and H_2_O molecules present in the environment can be trapped on the surface of n-type materials like ZnO to produce electron-trapping centers to slow down the transition time [44]. In the work reported herein the effect of H_2_O could also be amplified by the ability of CNF to scavenge H_2_O molecules from the environment.

**Figure 7:**
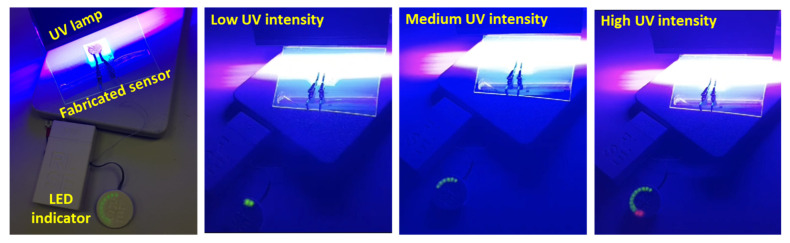
Demonstration of fabricated ZnO-NRs@CNF device as a UV sensor using a portable readout system with an LED indicator. The LEDs turn from green to red with increasing UV intensity levels. See Video S1, in Supplementary Materials for the full demonstration.

Figure 7 displays a demonstrative version using a circular LED array as an indicator, where the UV sensitivity of the fabricated ZnO-NRs@CNF device is displayed under varying UV intensity of the light source (0–10 W). As the UV intensity increases, more LEDs turn on and eventually turn from green to red at high intensity. In Video S1, Supplementary Materials, the demonstration of the fabricated device as a UV sensor is accomplished; as the UV light intensity increases more LEDs turn on as an indicator of increased sensitivity.

## 4. Conclusions

In summary, we have presented a scalable simple single-step approach for the simple and fast growth of ZnO NRs on the surface of CNFs. The growth of the ZnO NRs was strongly influenced by the presence of native/induced charges on the CNFs. While free ZnO in the solution was prevalent in the case of the enzymatic CNF and CNF with the lowest DS, the presence of CNF DS0.3 resulted in the preferential growth of ZnO NRs onto the fibrils. To assess the photoconductivity of the developed ZnO NRs/CNF composites obtained using CNF DS0.1 and CNF DS0.3, these composites were used to modify the surface of printed interdigitated electrode arrays. The ZnO NRs/CNF nanocomposites obtained with CNF DS0.3 showed high photoconductivity with fast ON/OFF switching under UV illumination. The results reported herein show that the fabricated nanocomposite structures have the potential to open new methods for the fabrication and development of advanced electronic devices including chemical/biosensors, solar cells, light-emitting diodes, photoelectrodes, UV/strain/humidity sensors, actuators, transistors, piezoelectric energy generators, capacitors, and batteries.

## Data Availability

The data that support the findings of this study are available upon reasonable request from the authors.

## Supplementary Materials

The following supporting information can be downloaded at https://www.mdpi.com/article/10.3390/nano13182611/s1.

## Abbreviations

Zinc oxide (ZnO), Cellulose nanofibers (CNFs), Nanorods (NRs), Ultraviolet (UV), Hexamethylene-tetramine (HMT), Scanning-electron-microscope (SEM), Fourier transform infrared spectroscopy (FTIR), Energy-dispersive X-ray spectroscopy (EDS), Degree of substitution (DS).
